# Identification of toxic mold species through Raman spectroscopy of fungal conidia

**DOI:** 10.1371/journal.pone.0242361

**Published:** 2020-11-23

**Authors:** Benjamin D. Strycker, Zehua Han, Zheng Duan, Blake Commer, Kai Wang, Brian D. Shaw, Alexei V. Sokolov, Marlan O. Scully

**Affiliations:** 1 Institute for Quantum Science and Engineering, Texas A&M University, College Station, Texas, United States of America; 2 Baylor University, Waco, Texas, United States of America; 3 Center for Optical and Electromagnetic Research, South China Academy of Advanced, Optoelectronics, South China Normal University, Guangzhou, China; 4 Department of Plant Pathology and Microbiology, Texas A&M University, College Station, Texas, United States of America; University of Nebraska-Lincoln, UNITED STATES

## Abstract

We use a 785 nm shifted excitation Raman difference (SERDS) technique to measure the Raman spectra of the conidia of 10 mold species of especial toxicological, medical, and industrial importance, including *Stachybotrys chartarum*, *Penicillium chrysogenum*, *Aspergillus fumigatus*, *Aspergillus flavus*, *Aspergillus oryzae*, *Aspergillus niger*, and others. We find that both the pure Raman and fluorescence signals support the hypothesis that for an excitation wavelength of 785 nm the Raman signal originates from the melanin pigments bound within the cell wall of the conidium. In addition, the major features of the pure Raman spectra group into profiles that we hypothesize may be due to differences in the complex melanin biosynthesis pathways. We then combine the Raman spectral data with neural network models to predict species classification with an accuracy above 99%. Finally, the Raman spectral data of all species investigated is made freely available for download and use.

## Introduction

Fungi are ubiquitous in both natural and man-made environments, and their effects upon the biosphere as well as human activity and health are both substantial and profound. For example, many fungi naturally form complex webs of mycorrhizal networks that facilitate plant-fungus symbiotic relationships through the exchange of nutrient and molecular resources [1–3]. The vast majority of plant species benefit from these cooperative dynamics [1]. Humans also benefit from fungi in numerous ways. Fungi have been used for millennia in the process of fermenting food products [4]. Traditional foods such as cheeses, soy sauce, miso, sonti, and tempeh owe their unique flavors to the molecules produced through fungal metabolism. These same fungal metabolic processes have found industrial use, as well, in producing mass quantities of lactic, citric, acetic, and other acids, in addition to antibiotics, vitamins, ethyl alcohol, amino acids, hormones, single-cell proteins, and fats [5]. The list is sure to grow longer as time passes. In addition to these positive impacts, fungi can also be deleterious to humans. Toxic molds growing in homes and work buildings can produce poisonous mycotoxins that exacerbate preexisting health conditions [6, 7]. In recent years within the United States, mold-related asthma has affected 4.6 million people for a total annual cost of $3.5 billion [8]. The costs of deleterious fungal growth are substantial in agriculture, as well. Every year, American agri-producers lose millions of dollars to mold exposure, infection, and damage [9]. Consequently, it is well known that harmful molds pose a significant threat to both public and economic health [10].

An important aspect of mitigating the damages due to deleterious mold growth is detection and identification. We envision the development of a mobile Raman spectroscopy scheme for timely and on-site identification and/or biological characterization. Several groups have contributed studies toward the realization of this goal. Ghosal *et al*. used spontaneous Raman micro-spectroscopy to measure and differentiate seven mold species [11]. Likewise, Farazkhorasani measured and differentiated several strains of *Aspergillus nidulans* (*A*. *nidulans*) [12], and several groups have reported results of Surface-Enhanced Raman Spectroscopy (SERS) for the same species [12, 13]. In a previous work, our group measured the Raman signature of *A*. *nidulans* using Coherent Anti-Stokes Raman Spectroscopy (CARS) an order of magnitude faster than spontaneous Raman techniques [14]. One disadvantage to doing so, however, is the comparative cost and complexity of a CARS setup in relation to a simpler, more robust, and initially more mobile spontaneous Raman scheme. Even so, we subsequently showed that even a simple spontaneous Raman scheme has its own pitfalls [15]. Using Shifted-Excitation Raman Difference Spectroscopy (SERDS), we separated the genuine Raman signal from spurious “fake” fine-scale fluorescence features that also appear in the spectrum and that a naïve analysis would misidentify as belonging to particular molecular vibration modes. We also showed that for an excitation wavelength of 785 nm the Raman signal of *A*. *nidulans* conidia originates from pigment molecules within the cell wall [15]. While this suggests that the same is likely to be true for conidia of other species, as well, it begs the question of whether 785 nm excitation will be able to yield a useful Raman signal in all cases, since the pigmentation of mold species is highly variable.

For the interrogation of biological samples, 785 nm excitation is often considered to be optimal, since it reduces the obscuring fluorescence excited by shorter wavelengths and yet may still be detected with conventional charge-coupled device (CCD) technology, which has limited sensitivity for longer infrared wavelengths [16]. Previous works demonstrated that a spontaneous 785 nm Raman scheme can characterize a handful of readily available and benign mold species [11, 12, 15]. But can a spontaneous 785 nm Raman scheme be used to characterize the particular mold species that are known to be of especial toxicological, medical, or industrial importance? And, if so, what kind of technique is required to accurately identify and differentiate them? These are the questions that guide the present work. Here, we analyze the Raman spectra of ten mold species using the SERDS technique and quantify the level of inter-species differentiation in order to determine necessary experimental practices.

The mold species have been chosen for their medical, industrial, and scientific interest. They are species which have a large impact on human life and activity. *Stachybotrys chartarum* (*S*. *chartarum*), the infamous “black mold,” has received increased popular attention in recent decades on account of the human health effects of the mycotoxins that it produces [17, 18]. It is a common contaminant of damp buildings [19]. *Penicillium chrysogenum* (*P*. *chrysogenum*) is used in the bulk industrial production of penicillin [20]. It is also a common contaminant of damp buildings [19]. Eight different species from the genus *Aspergillus* were selected in order to assess whether closely related species could be differentiated using SERDS [21]. *Aspergillus fumigatus* (*A*. *fumigatus*), whose airborne conidia are among the most prevalent worldwide, is the fungus responsible for 90% of aspergillosis cases, the infectious colonization of the lungs in immunocompromised individuals [22–24]. *Aspergillus niger* (*A*. *niger*) is an important industrial fungus used in the production of citric acid and of enzymes such as amylases (for example glucoamylase, which is used in producing corn syrup), pectinases (used to clarify cider and wine), and proteases (used in detergents) [25–27]. It may also cause aspergillosis [22]. *Aspergillus oryzae* (*A*. *oryzae*) has been used for thousands of years in food and beverage fermentation. Its preparation is commonly known as koji. Among other products, it is used to produce sake, soy sauce, and miso on industrial scales, each of which is worth billions of dollars annually [4, 28]. *Aspergillus flavus* (*A*. *flavus*) is of great economic importance as a pathogen in pre- and post-harvest agricultural crops such as corn, peanuts, cotton, and treenuts [29]. It produces Aflatoxin B_1_, the most potent naturally occurring carcinogen and one of the few mycotoxins to have been developed as a biological weapon [30]. *A*. *flavus* may also cause aspergillosis [22]. *Aspergillus terreus* (*A*. *terreus*) is known to produce several anticancer bioactive compounds and is a source of lovastatin, which is used therapeutically to treat lipid disorders [31]. It may also cause aspergillosis [22]. *Aspergillus clavatus* (*A*. *clavatus*) is an allergenic species that causes hypersensitivity pneumonitis, also known as “malt workers’ lung” [31]. *Aspergillus versicolor* (*A*. *versicolor*) is another common contaminant of damp buildings [19]. Finally, *A*. *nidulans* has been studied extensively since the 1940’s as a scientific model organism [32]. It may also cause aspergillosis [22].

In the work described here, we investigate which species may successfully be identified through Raman spectroscopy using 785 nm excitation. In addition, in view of the medical and economic importance of these species, we make their averaged Raman spectra data freely available for download and use in the Supporting Information.

## Methods and materials

### Sample preparation

Strains used include *A*. *nidulans* (FGSC A4), *A*. *niger* (CBS 513.88), *A*. *flavus* (NRRL 3557), *A*. *fumigatus* (Af293), *A*. *oryzae* (ATCC 14895), *A*. *versicolor* (CBS 795.97), *A*. *clavatus* (NRRL1), *A*. *terreus* (NIH2624), *P*. *chrysogenum* (ATCC 10106), and *S*. *chartarum* (ATCC 201867). Strains were plated in triplicate onto minimal media (MM) [33] and incubated at 30 °C under continuous light. After 7 days, cultures were flooded with 2 mL sterile water and spores were dislodged by scraping with a sterile glass rod. The spore suspensions were then washed a minimum of three times each by centrifuging for 1 min at 13000 rpm, as previously described [14]. Spore suspensions at a final concentration of 1 × 10^6^ mL^-1^ were stored at 4 °C when not in use.

### SERDS experiments

**Fig 1:**
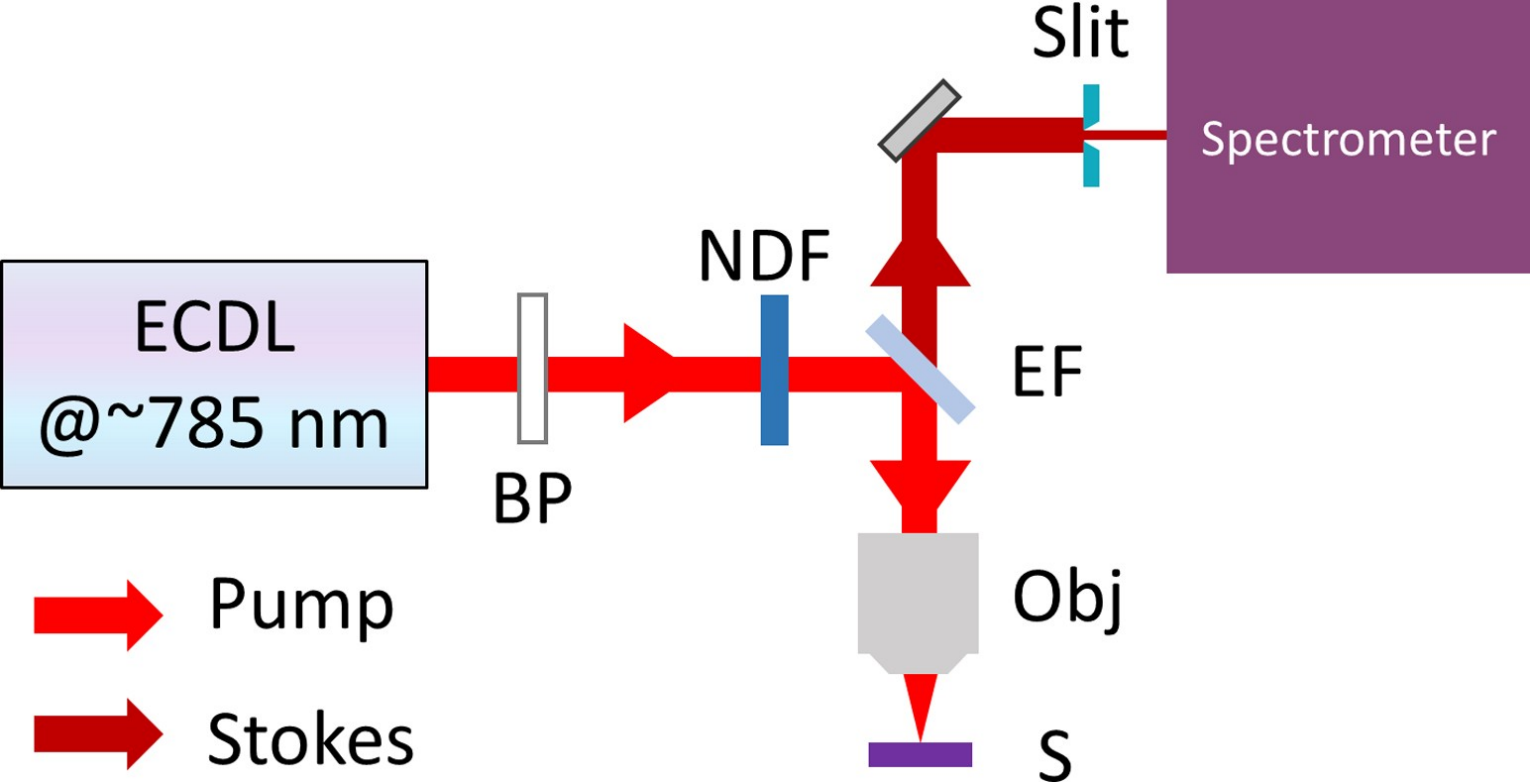
Simplified schematic of SERDS experimental layout. ECDL: external cavity diode laser; BP: bandpass filter; NDF: neutral density filter; EF: edge filter; Obj: microscope objective; S: sample.

The SERDS experimental setup is the same used for our previous work [15] (see Fig 1). Briefly, ~ 6 μL of the spore suspension was deposited on a fused silica window (WG 41010, Thorlabs) and allowed to evaporate over the course of several hours, leaving the spores as a deposit on the surface. We then placed the sample under a Raman microscope (LabRAM HR Evolution, Horiba) for Raman interrogation using light emitted from a tunable laser working at ~ 785 nm, which was focused on the sample through a 100x objective (HCX PL Fluotar, N.A. 0.75). The backscattered signals from the sample were collected by the same objective. Two slightly different excitation wavelengths were applied to generate two spectra for each spore. Each recorded spectrum was the average of 12 5-second acquisitions, for a total integration time of 60 seconds. In general, each spectrum contains elements resulting from both Raman and fluorescence mechanisms. However, genuine Raman spectral features shift in unison with the excitation wavelength, while spectral features resulting from fluorescence mechanisms are independent of these shifts. The pure Raman spectrum can be retrieved by integrating the difference spectrum according to well-established SERDS protocols [34–40]. Here, post-processing of the raw data in order to retrieve the pure Raman spectra was similar to that described in our previous work [15] and is described in greater detail in the S1 Appendix.

### Neural network

In order to characterize the accuracy with which the raw and Raman spectra can be used to classify single spores as belonging to particular species, three neural network (NN) models were developed and tested using the TensorFlow software package [41]. Model 1 consisted of two sequential dense layers. The first layer was composed of 100 neurons activated by rectified linear unit (ReLU) functions. The output layer was composed of 8 neurons. Model 2 consisted of three sequential dense layers. The first two layers were composed of 100 neurons activated by ReLU functions. The output layer was composed of 8 neurons. Model 3 consisted in four sequential dense layers. The first three layers were composed of 100 neurons activated by ReLU functions. The output layer was composed of 8 neurons. All models were compiled using Adam optimization.

The data pool included the spectra from 100 spores each from *A*. *nidulans*, *A*. *flavus*, *A*. *oryzae*, *A*. *versicolor*, *A*. *fumigatus*, *A*. *niger*, *S*. *chartarum*, and *P*. *chrysogenum*, for a total of 800 spores. In order to evaluate the accuracy with which a single spore spectrum could be classified as belonging to one of the above eight species, the spectrum of each spore was in turn subtracted from the data pool while the remaining 799 spore spectra were used to train the NN. Evaluation of all 800 spores therefore required 800 separate instances of NN training with a corresponding 800 separate instances of applying the NN to the classification of a single spore spectrum. Each instance of training consisted of 20 epochs for which the order of the training spectra was randomly shuffled so as to reduce the dependence of the model on the sequence with which the data was measured.

The above protocol for training and classifying each of 800 spore spectra in turn was repeated 10 times to accumulate average statistics. Each NN model, therefore, was subjected to 8000 total instances of training and evaluation.

## Results and discussion

**Fig 2:**
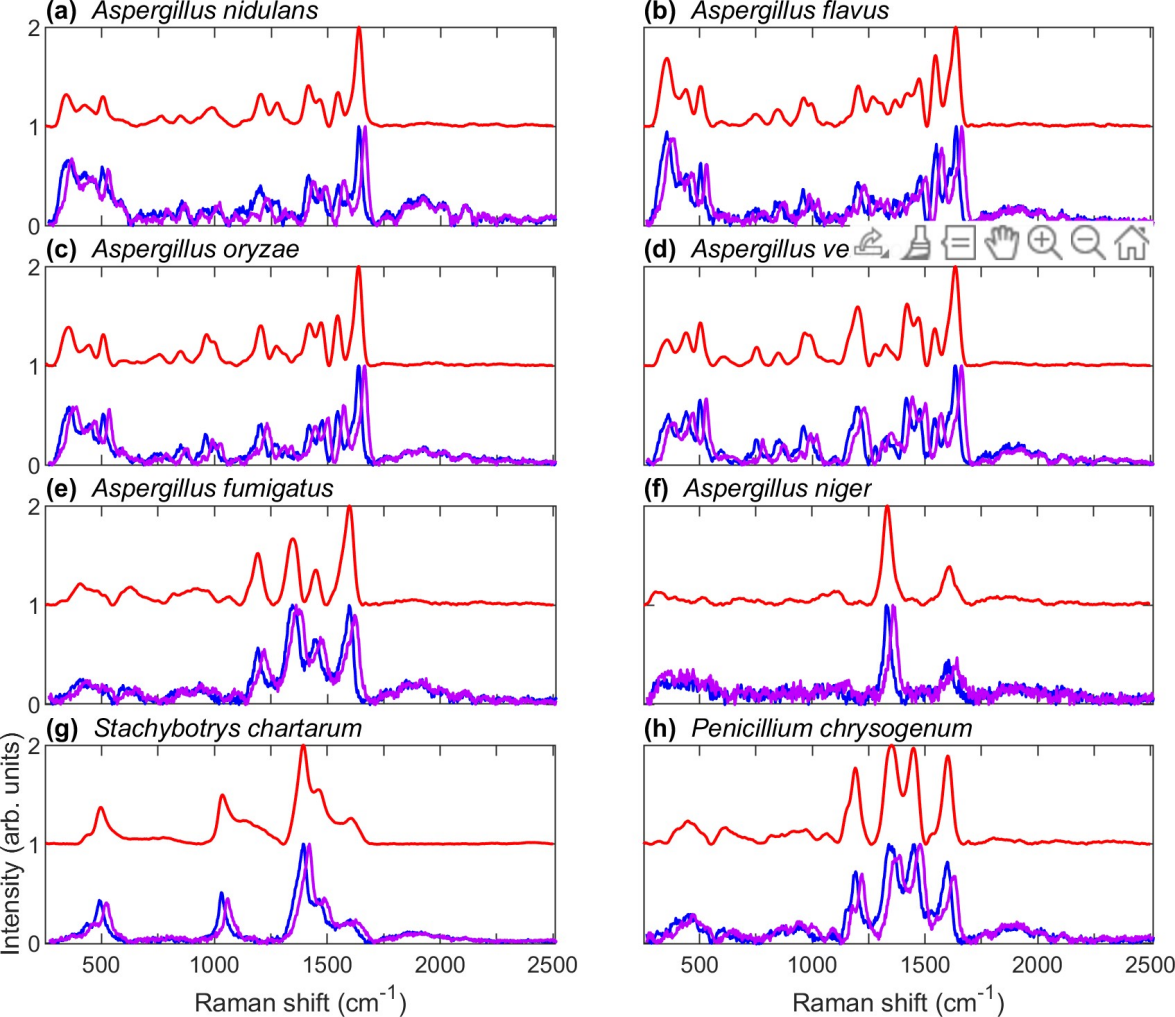
Single spore Raman spectra of species that yielded usable signals. The blue and purple curves correspond to Asymmetric Least-Squares (AsLS) [42] background-subtracted raw spectra excited by two slightly different frequencies, while the red curves correspond to the reconstructed, pure Raman spectra obtained through the SERDS protocol outlined in the S1 Appendix.

We report that, using a SERDS technique with an excitation wavelength of ~785 nm, conidia from neither *A*. *clavatus* nor *A*. *terreus* yielded a Raman signal of sufficient strength to be discernable above fluorescence. The spectra of these species are included in the S2 Appendix. Fig 2 shows single spore Raman spectra of the species which yielded usable signals. The blue and purple curves correspond to Asymmetric Least-Squares (AsLS) [42] background-subtracted raw signals excited by two slightly different wavelengths. As can be seen, the genuine Raman features shift with excitation wavelength, while features resulting from fluorescence remain unaffected. The red curves correspond to the reconstructed, pure Raman spectra obtained through the SERDS protocol outlined in the S1 Appendix.

**Fig 3:**
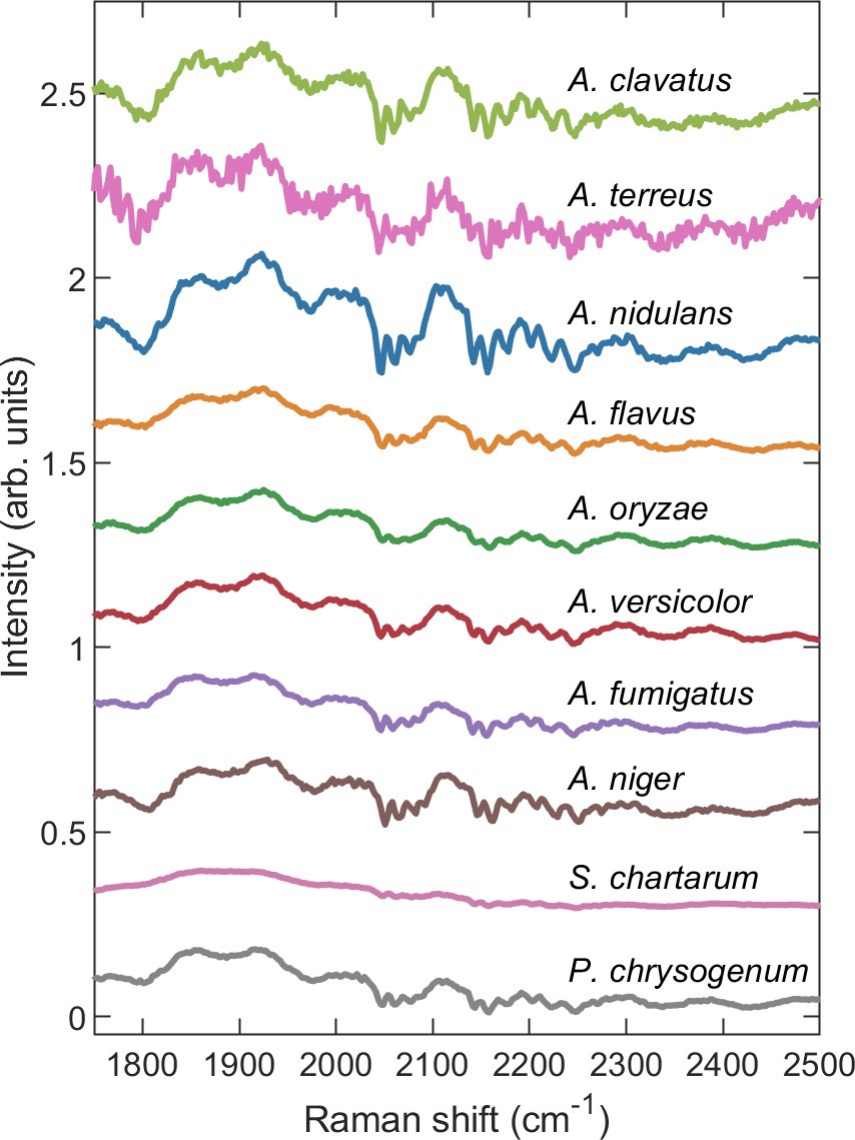
Averaged AsLS background-subtracted raw spectra showing fine-scale fluorescence features in the range ~1750 to 2500 cm^-1^.

The region between approximately 1750 and 2500 cm^-1^ contains fine-scale fluorescence features common to all species. This is shown more fully in Fig 3. The curves are averages of AsLS [42] background-subtracted raw spectra obtained from multiple spores, corresponding to many different measurements of the blue curves shown in Fig 2. For *A*. *clavatus* and *A*. *terreus*, the number of different spores used was *n* = 50 and *n* = 10, respectively. For all other species, the number of different spores used was *n* = 100. In our previous work, we showed that for *A*. *nidulans* the Raman signal excited by a 785 nm laser originates in pigment molecules located within the cell wall [15]. Since the pool of species investigated here exhibits widely different melanin pigments, both the variety of major Raman features in Fig 2 and the similarity of the fine-scale fluorescence features in Fig 3 support the hypothesis that at 785 nm the Raman signal originates in melanin pigments within the cell wall while the fine-scale fluorescence signal originates from constituents of the cell wall itself, regardless of species. In fungi, the cell wall is composed of a highly cross-linked biopolymer formed from glucans, chitin and chitosan, mannans and/or galactomannans, and glycoproteins [43]. Further investigation is required to determine which of these molecular constituents is responsible for the fine-scale fluorescence features shown in Fig 3. It should be noted that *S*. *chartarum* does not exhibit these fine-scale fluorescence features or, if it does, exhibits them only weakly. Of the species investigated, *S*. *chartarum* is the only one which produces conidial spores covered in a layer of slime, which may possibly serve to suppress the mechanism responsible for the fine-scale fluorescence exhibited in other species. We have hypothesized elsewhere [15] that the fine-scale fluorescence features may be facilitated by the formation of long-lived molecular cages within the biopolymer of the cell wall, thus partially shielding fluorophores from the substantial inhomogeneous broadening they would otherwise experience in a different environment [44, 45].

The hypothesis that the Raman signal originates in melanin pigments within the cell wall is further supported through an investigation of the melanin biosynthesis pathway of each species. Fungal melanins are negatively charged, hydrophobic, and of high molecular weight. Melanin functions to protect the fungal microorganism from harmful environmental conditions such as ultraviolet light-induced and oxidant-mediated damages, temperature extremes, hydrolytic enzymes, heavy metal toxicity, and anti-microbial drugs [46]. In conidial spores, they are concentrated and bound within the cell wall. Because of their integration within the cross-linked network that constitutes the cell wall, the molecular structures of fungal melanins remain notoriously recalcitrant to characterization [46, 47]. Nevertheless, two main pathways for fungal melanin biosynthesis have been identified. One pathway begins with malonyl-CoA and leads to the polymerization of 1,8-dihydroxynapthalene (DHN), resulting in DHN-melanin. An alternative pathway begins with L-dopa or tyrosine and leads to polymerization of dihydroxyindoles, resulting in DOPA-melanin [46].

To our knowledge, the melanin biosynthesis pathways of only six of the ten species under investigation have been studied. *A*. *terreus* possesses a DHN-melanin pathway [48], as does *A*. *fumigatus* [49, 50]. The biosynthesis pathway classifications of the fungi *A*. *nidulans*, *A*. *oryzae*, *A*. *flavus*, and *A*. *niger* remain uncertain [47]. It has long been recognized that these species possess gene orthologues encoding Polyketide Synthases (PKSs) that in *A*. *fumigatus* are responsible for the production of DHN melanin. However, their role in conidial pigmentation for *A*. *niger* and *A*. *flavus* is doubtful [47]. *A*. *nidulans*, *A*. *oryzae*, *A*. *flavus*, and *A*. *niger* have all at one time been classified as DOPA melanin producers [48, 50, 51]. In *A*. *oryzae*, culturing in the presence of the DOPA precursor tyrosine results in the production of DOPA melanin [52]. DOPA pathway inhibitors have prevented pigmentation in *A*. *nidulans* [51], *A*. *flavus* [48], and *A*. *niger* [48], although subsequent work seemed to cast doubt on these results [53]. It has been surmised that the black pigment of *A*. *niger*, known as aspergillin, is actually a polymerization of two different pigments, one brown and one green. Consequently, the pigmentation of *A*. *niger* is acknowledged to be complex and is still under investigation [47]. Regardless, what is abundantly clear from the literature is that *A*. *nidulans*, *A*. *flavus*, and *A*. *niger* do not respond to DHN pathway inhibitors [48, 51, 53].

**Fig 4:**
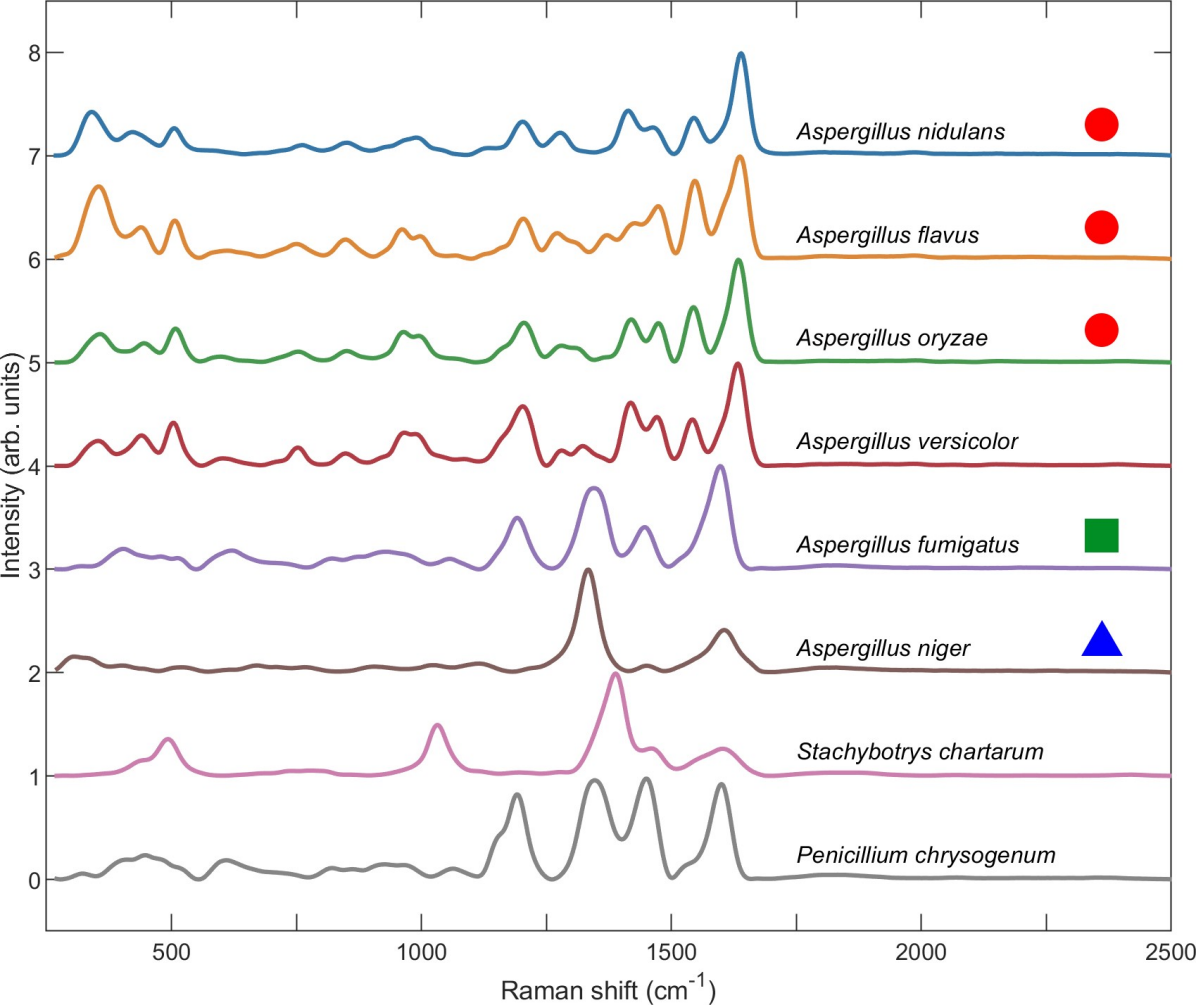
Pure Raman spectra of various species retrieved from the SERDS protocol outlined in the S1 Appendix. Each spectrum is the average of the SERDS spectra retrieved from *n* = 100 individual spores. The red circles indicate that a species either produces DOPA melanin when cultured in the presence of tyrosine (*A*. *oryzae*) or remains unaffected by DHN pathway inhibitors (*A*. *nidulans* and *A*. *flavus*). The green square indicates that conidial pigmentation is due to DHN melanin (*A*. *fumigatus*). The blue triangle indicates that, while *A*. *niger* is not affected by DHN pathway inhibitors, it is thought to polymerize two pigments [47, 48].

It can be seen in Fig 4 that the melanin biosynthesis pathway of a species correlates with major features in its reconstructed, pure Raman spectrum. The top four spectra are remarkably similar. As stated above, three of these species have been shown either to produce DOPA melanin when cultured in the presence of tyrosine (*A*. *oryzae*) or remain unaffected by DHN pathway inhibitors (*A*. *nidulans* and *A*. *flavus*). It is therefore likely that *A*. *versicolor* is not affected by DHN pathway inhibitors, as well, although to our knowledge no study on the melanin biosynthesis pathway of this species has been completed. It can also be seen that, although *A*. *niger* also remains unaffected by DHN pathway inhibitors, the unusual complexity of its pigmentation is fully reflected in the vast difference of its Raman spectrum from the other Aspergillus species. We point out that *A*. *fumigatus*, whose conidial pigmentation is produced through the DHN pathway, exhibits a different Raman spectrum from the rest of the Aspergillus species. The similarity between the Raman spectra of *A*. *fumigatus* and *P*. *chrysogenum* leads us to suppose that DHN pathway inhibitors will be able to prevent pigmentation in *P*. *chrysogenum*, although to our knowledge the melanin biosynthesis pathway of this species has not been studied. Neither has the melanin biosynthesis pathway of *S*. *chartarum* been studied, but its Raman spectrum suggests that possibly, like *A*. *niger*, its biosynthesis pathway may be more complex than other species. Even the spectra of *A*. *terreus*, which did not yield a Raman signal of sufficient strength to be reliably measured, fits into this paradigm. *A*. *terreus* is unique among Aspergilli in that it does not use the commonly found Polyketide Synthases (PKSs) for pigment biosynthesis, as do other Aspergillus species. Instead, *A*. *terreus* pigment biosynthesis occurs through enzymes similar to NonRibosomal-Peptide Synthetases (NRPSs), namely MelA, the only such enzymes found thus far [47, 54]. Unfortunately, the literature contains little information on melanin biosynthesis pathway for *A*. *clavatus*.

The fact that a species’ melanin biosynthesis pathway correlates with major features in its Raman spectrum is perhaps not surprising, given the above evidence that the Raman signal originates from melanin within the cell wall. Similar melanin biosynthesis pathways begin and end with similar molecules, each possessing similar vibrational modes which, upon optical excitation, result in similar Raman spectra. The minor differences in the location and amplitude of various peaks in these Raman spectra may be reflective of differences in (1) the structure of molecular precursors, (2) the polymerization enzymes responsible for melanin production, (3) and/or the ratios of molecular components of the cell wall into which the pigments have been integrated. The remarkable similarity between the Raman spectra of some of the species under investigation begs the question of whether their Raman spectra may be used to successfully identify and differentiate them. It is to this question that we now turn to next.

Neural Networks (NN) are powerful tools for the analysis of multidimensional data [55, 56]. Indeed, they are often able to distinguish data patterns that remain invisible to unaided human perception. Consequently, they are increasingly being used across many fields, including cancer diagnosis [57, 58], communications [59], cyber security [60], earthquake detection and location [61], and construction automation [62]. Here, we use a NN model to assess the accuracy with which a spectrum from a single spore may be used to classify the species to which that spore belongs. We point out that our goal is not the development of an optimized model, but rather the characterization of a baseline model from which further improvements can be made.

We tested three different NN models of increasing complexity as outlined in **Methods and Materials**, consisting of two, three, and four sequential layers, respectively. Two different types of spectra were used for single spore species characterization: (1) the pure Raman spectrum retrieved through the SERDS protocol and (2) the AsLS background-subtracted raw spectrum. These spectra types correspond to the red and blue curves shown in Fig 2, respectively. The NN classification accuracy for spores of eight different species is listed in Table 1. We point out that in all cases the classification accuracy never drops below 99%. If significance is attached to the approximately 1% error, it can be seen that *A*. *versicolor* poses the greatest challenge to classification, since it can exhibit a widely varying spore color. In addition, it can be seen that in all cases the raw spectrum exhibits greater total classification accuracy than the pure Raman spectrum. This may be due to an amount of spectral information contained in the fluorescence response that is excluded from the pure Raman signal. Finally, as the NN model complexity increases with the number of layers, the classification accuracy of the raw spectrum decreases, which is indicative of overfitting. Consequently, the greatest total classification accuracy of 99.975% is achieved with a simple two-layer NN model using raw measured spectra. Regardless, it is evident that remarkable classification accuracy can be achieved even for species that exhibit spectra as similar as those shown in Fig 4.

**Table 1:** Percent accuracy with which single spore spectra from a given species may be classified using the Neural Network (NN) models described in methods and materials.

Accuracy of Classification (%)											
*Model*	*Layers*	*Measurement*	*A*. *nidulans*	*A*. *flavus*	*A*. *oryzae*	*A*. *versicolor*	*A*. *fumigatus*	*A*. *niger*	*S*. *chartarum*	*P*. *chrysogenum*	*Total*
1	2	Raman	100	100	100	99.7	100	100	100	99.2	99.8625
		Raw	100	100	100	99.8	100	100	100	100	99.975
2	3	Raman	100	100	100	99.9	100	100	100	99.6	99.9375
		Raw	100	100	100	99.6	100	100	100	100	99.95
3	4	Raman	100	100	99.9	99.6	100	100	100	99.7	99.9
		Raw	99.9	100	100	99.7	99.9	100	100	100	99.9375

## Conclusion

We have used a 785 nm SERDS technique to measure the Raman spectra of single conidial spores of 10 mold species of especial toxicological, medical, and industrial importance. In conjunction with our previous work [15], both the pure Raman and fluorescence signals support the hypothesis that the Raman signal originates from the melanin pigments bound within the highly cross-linked cell wall of the conidium. The major features of the Raman spectrum correlate with the melanin bio-synthesis pathway: species that either produce DOPA melanin when cultured in the presence of tyrosine or remain unaffected by DHN pathway inhibitors exhibit similar Raman spectra, which differ in turn from the Raman spectra of species that produce DHN melanin. Species with a complex melanin bio-synthesis pathway exhibit even more varying Raman spectra. These Raman spectral relationships lead us to predict that *A*. *versicolor* will not be affected by DHN pathway inhibitors, while in *P*. *chrysogenum* DHN pathway inhibitors may prevent conidial pigmentation. In the future we expect that Raman spectroscopy will become a powerful tool in the characterization of fungal melanins and their biosynthetic pathways when used in conjunction with genetic and chemical inhibitor approaches. Recently Pacelli et al. [63] demonstrated through a multidisciplinary approach that the fungus *Cryomyces antarcticus* produces both DHN and DOPA melanin in differing amounts. It is possible that the same may be true of other species, as well. Our work demonstrates that Raman spectroscopy can be used in such a multidisciplinary approach to great effect.

We highlight the fact that two of the investigated species, *A*. *terreus* and *A*. *clavatus*, did not exhibit Raman spectra that could be measured reliably over against the fluorescence response. As noted above, this is likely due to the characteristics of the melanin molecules within the cell wall. In the case of *A*. *terreus*, its unique biosynthesis pathway makes it an exception among mold species. The melanin biosynthesis pathway of *A*. *clavatus*, to our knowledge, remains unknown. Regardless, these facts must be taken into account in the design of a system for Raman identification of mold species. While spontaneous Raman techniques offer simplicity of implementation, a more complex coherent Raman characterization technique, such as CARS [14], may be able to successfully measure a Raman spectrum where a spontaneous Raman technique fails, since coherent Raman processes give a much larger signal. The pros and cons of each approach must be weighed.

In addition, we found that for those species which exhibited measurable Raman spectra, a species classification accuracy above 99% was easily achieved with simple NN models. This is highly encouraging and bodes well for the success of Raman identification of mold species, even for those species whose Raman spectra exhibit a high degree of similarity. Moreover, the implementation of a fluorescence-suppression technique, in this case SERDS, is not necessary for successful and highly accurate species classification, since our results indicate that the presence of fine-scale fluorescence features in the spectrum does not detract from the information contained in the Raman response. Raw measured spectra even exhibited a slightly greater classification accuracy than those spectra calculated through a SERDS protocol, due to minor variations in the fluorescence response that aid in classification. Consequently, while fluorescence suppression techniques are required in order to retrieve an accurate Raman spectrum, for identification purposes alone a simple “point and shoot” technique will still be highly accurate.

The above results demonstrate the practicality of Raman spectroscopy for the identification of toxic and industrial mold species. Full implementation, however, depends on the development of a mobile Raman microspectrometer. Current commercial handheld spectrometers will not suffice, since analysis of mold conidia requires sensitive control of sample position, laser power, and spectral integration settings, all of which these devices do not offer. Commercial Raman microspectrometers, however, which do offer sufficient experimental finesse for the application, are bulky and cannot easily be transported. Portability would require miniaturization, and such a product does not currently exist on the market. Nevertheless, the main components of a portable Raman microspectrometer, namely 1) a miniaturized intensified CCD spectrometer, 2) a compact variable-power Raman laser source, and 3) a finely-tuned microscope stage, all exist commercially as independent products. What remains to be done is only a matter of assembling them into an integrated device, which we intend to accomplish in our future research.

Finally, we wish to emphasize that this is the first time the Raman spectra of the toxic *S*. *chartarum* and infectious *A*. *fumigatus* have been published. We have also measured the Raman spectra of the carcinogenic *A*. *flavus*, medicinal *P*. *chrysogenum*, and industrial *A*. *oryzae* and *A*. *niger*. Other important species are included, as well. Both the pure Raman and raw spectra of all these species are made freely available for download and use in the Supporting Information and online [64].

## Supporting information

S1 Appendix Protocol for calculating retrieved pure Raman spectra. (PDF)

S2 Appendix Spectral plots of *Aspergillus clavatus* and *Aspergillus terreus*. (PDF)

S1 Dataset *Aspergillus nidulans*. (XLSX)

S2 Dataset *Aspergillus flavus*. (XLSX)

S3 Dataset *Aspergillus oryzae*. (XLSX)

S4 Dataset *Aspergillus versicolor*. (XLSX)

S5 Dataset *Aspergillus fumigatus*. (XLSX)

S6 Dataset *Aspergillus niger*. (XLSX)

S7 Dataset *Stachybotrys chartarum*. (XLSX)

S8 Dataset *Penicillium chrysogenum*. (XLSX)
