# Supplementary material for: Identification of toxic mold species through Raman spectroscopy of fungal conidia
Source: PLoS One. 2020 Nov 23;15(11):e0242361. doi: 10.1371/journal.pone.0242361 (PMC7682877; doi:10.1371/journal.pone.0242361)
Supplement: S1 Appendix — (PDF) [file pone.0242361.s001.pdf]

S1 Appendix

Measurement and calculation of the pure, retrieved Raman spectrum was similar to our previous work [1], which was based on the SERDS protocol outlined in [2]. For each individual spore, two spectra were measured at slightly shifted frequencies at a laser power on the order of a mW. Table S1 lists for each species the number *n* of spores measured, the average wavelengths corresponding to each of the two excitation frequencies λ_1 and λ_2, and the average change in frequency $\Delta\nu$ between the two excitation frequencies.

**Species**	***n***	$\overline{\boldsymbol{\lambda_1}}$ (nm)	$\overline{\boldsymbol{\lambda_2}}$ (nm)	$\overline{\boldsymbol{\Delta\nu}}$ (cm^{-1})
A. clavatus	50	784.3290 ± 0.0050	785.8767 ± 0.0015	25.1103 ± 0.0865
A. terreus	10	784.2203 ± 0.0007	785.8776 ± 0.0007	26.8914 ± 0.0078
A. nidulans	100	784.3275 ± 0.0061	785.8740 ± 0.0249	25.0895 ± 0.4436
A. flavus	100	784.2142 ± 0.0055	785.8742 ± 0.0034	26.9356 ± 0.1015
A. oryzae	100	784.2187 ± 0.0024	785.8796 ± 0.0047	26.9490 ± 0.0808
A. versicolor	100	784.2232 ± 0.0036	785.8832 ± 0.0047	26.9341 ± 0.0712
A. fumigatus	100	784.2269 ± 0.0015	785.7724 ± 0.0072	25.0794 ± 0.1201
A. niger	100	784.1070 ± 0.0023	785.9879 ± 0.0025	30.5193 ± 0.0487
S. chartarum	100	784.1964 ± 0.0354	785.8556 ± 0.0367	26.9236 ± 0.1313
P. chrysogenum	100	784.2274 ± 0.0018	785.8887 ± 0.0012	26.9554 ± 0.0343

Table S1. Average experimental parameters for SERDS measurements.

Although Table S1 lists average experimental parameters, in practice the pure, retrieved Raman spectrum was calculated for each spore on an individual basis. Our procedure is based on that of [3] and is as follows, using a single *A. nidulans* spore for illustration:

First, the raw measured spectra shown in Figure S1(a) were z-normalized [3], resulting in the spectra shown in Figure S1(b). The spectrum corresponding to excitation wavelength λ_2 (purple in Figure S1(b)) was subtracted from the spectrum corresponding to λ_1 (blue in Figure S1(b)), yielding the red difference spectrum in Figure S1(c). The black trendline in Figure S1(c) was found using a 2nd order nonparametric LOWESS smoothing algorithm spanning 250 datapoints with three iterations (Matlab, mslowess [4]), which was subtracted to yield the baseline-corrected difference spectrum in Figure S1(d). The cumulative integral of this curve was taken (Matlab, cumtrapz [5]), which is shown in red in Figure S1(e) and which corresponds to the pure, retrieved Raman spectrum.

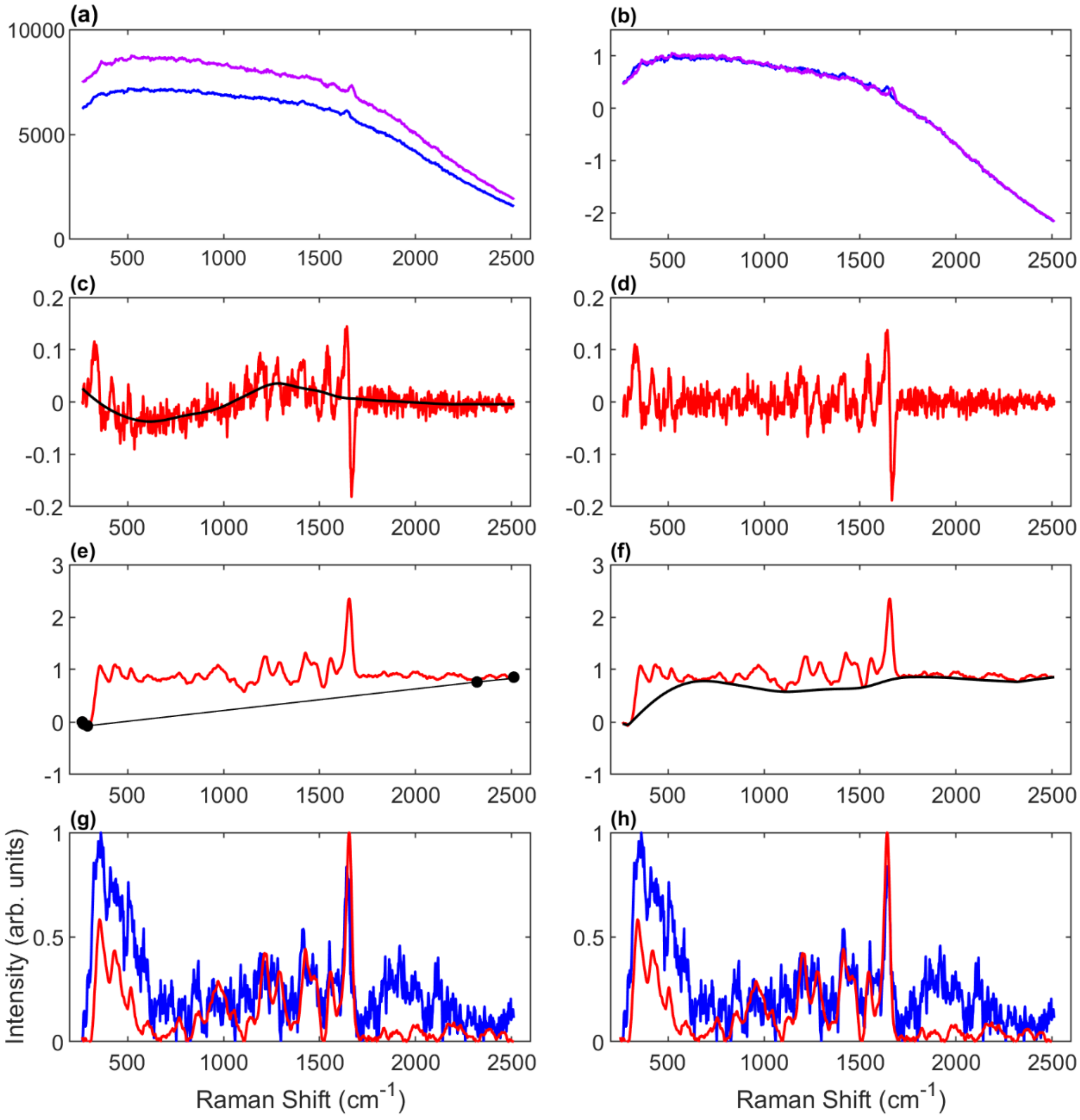

Figure S1. SERDS protocol applied to spectral measurements from a single *A. nidulans* spore. (a): Raw measured spectra corresponding to excitation wavelengths λ_1 (blue) and λ_2 (purple). (b): The z-normalized spectra in (a). (c): The difference spectrum (red) of the spectra in (b), with a trendline (black). (d): The trendline-subtracted difference spectrum. (e): The cumulatively integrated difference spectrum in (d) (red). Also shown are the endpoints of the largest sub-intervals of downward concavity (black dots), along with the lines connecting these endpoints. (f): The integrated difference spectrum (red) along with the final background curve (black). (g): A normalized pure, retrieved Raman spectrum (red) along with a normalized AsLS background-subtracted raw spectrum generated from the blue λ_1 curve in (b) (blue). (h): The final normalized pure, retrieved Raman spectrum that has been shifted by $-\Delta\nu/2$ (red) with a normalized AsLS background-subtracted raw spectrum generated from the blue λ_1 curve in (b) (blue).

A background-subtraction algorithm is commonly applied at this stage. Gebrekidan *et al*. [3] showed that a piece-wise background-subtraction algorithm applied over sub-intervals defined by local minima yields better results than the same algorithm applied over the whole curve at once. However, for our data we found that sub-intervals based on downward concavity rather than local minima yielded better results. An interval of a curve is concave down if and only if the line joining the endpoints of the interval lies on or below the curve [6]. As such, we sub-divided our curves into the largest possible intervals of downward concavity, that is, the largest intervals for which the line joining the endpoints of that interval lies on or below the curve. This is illustrated in Figure S1(e). The black dots mark the endpoints of the largest intervals of downward concavity, while the lines joining these endpoints are displayed for illustration.

An Asymmetric Least-Squares (AsLS) background-subtraction algorithm [7] was then applied to each sub-interval separately (with parameters p = 10^{-4}, lambda = 10^3). The values at the common endpoints of the sub-intervals of the generated background curve were averaged. The sub-intervals of the background curve were then concatenated, and to remove any lingering discontinuities both a 1σ Hampel filter spanning 11 datapoints (Matlab, hampel [8]) as well as a 2nd order nonparametric LOWESS smoothing algorithm spanning 10 datapoints with three iterations was applied (Matlab, mslowess [4]). The final background curve is shown in black in Figure S1(f). When subtracted from the red, integrated difference curve, it yields the background-subtracted pure, retrieved Raman spectrum shown in red in Figure S1(g), which has been normalized according to peak intensity. Also shown in blue in Figure S1(g) is a normalized background-subtracted raw spectrum, which was generated through simple application of an AsLS algorithm [7] (p = 10^{-4}, lambda = 10^3) to the blue z-normalized λ_1 spectrum in Figure S1(b).

The last step of the SERDS protocol is to shift the pure, retrieved Raman spectrum by $-\Delta\nu/2$ (see [2]). The final result is shown in red in Figure S1(h), along with an AsLS background-subtracted raw λ_1 spectrum shown in blue for comparison.

Again, the above procedure was applied on an individual basis to retrieve the pure Raman spectrum of each spore of a given species. Since for each spore the final shift by $-\Delta\nu/2$ is variable, 1D interpolation at common abscissae was required in order to generate the average spectra shown in Figure 4 of the main manuscript. This was accomplished through a cubic spline method using not-a-knot end conditions (Matlab, interp1 [9]).

References

1. Han Z, Strycker BD, Commer B, Wang K, Shaw BD, Scully MO, et al. Molecular origin of the Raman signal from Aspergillus nidulans conidia and observation of fluorescence vibrational structure at room temperature. Sci Rep. 2020;10:5428.

2. Zhao J, Carrabba MM, Allen FS. Automated fluorescence rejection using shifted excitation Raman difference spectroscopy. Appl Spectrosc. 2002;56(7):834–45.

3. Gebrekidan MT, Knipfer C, Stelzle F, Popp J, Will S, Braeuer A. A shifted-excitation Raman difference spectroscopy (SERDS) evaluation strategy for the efficient isolation of Raman spectra from extreme fluorescence interference. J Raman Spectrosc. 2016;47(2):198–209.

4. mslowess. The MathWorks, Inc.; 2008.

5. cumtrapz. The MathWorks, Inc.; 2017.

6. Avriel M, Diewert WE, Schaible S, Zang I. Generalized Concavity. Society for Industrial and Applied Mathematics; 2010.

7. Eilers PH, Boelens HF. Baseline correction with asymmetric least squares smoothing. Leiden Univ Med Cent Rep. 2005;1:5.

8. hampel. The MathWorks, Inc.; 2015.

9. interp1. The MathWorks, Inc.; 2018.
