# Supplementary material for: Identification of toxic mold species through Raman spectroscopy of fungal conidia
Source: PLoS One. 2020 Nov 23;15(11):e0242361. doi: 10.1371/journal.pone.0242361 (PMC7682877; doi:10.1371/journal.pone.0242361)
Supplement: S2 Appendix — (PDF) [file pone.0242361.s002.pdf]

S2 Appendix

A total of $n = 50$ and $n = 10$ spores were evaluated for *Aspergillus clavatus* and *Aspergillus terreus*, respectively. While in principle a retrieved, pure Raman spectrum can always be calculated from two raw spectra measured using the SERDS technique, in the case of these two species any pure Raman features did not exhibit sufficient signal-to-noise ratio to be reliably distinguished from minor spectral artefacts. For this reason we did not include the Raman spectra of *A. clavatus* and *A. terreus* in the main manuscript. They are reproduced here for the reader's sake with the understanding that any apparent spectral features may or may not correspond to actual molecular vibration modes.

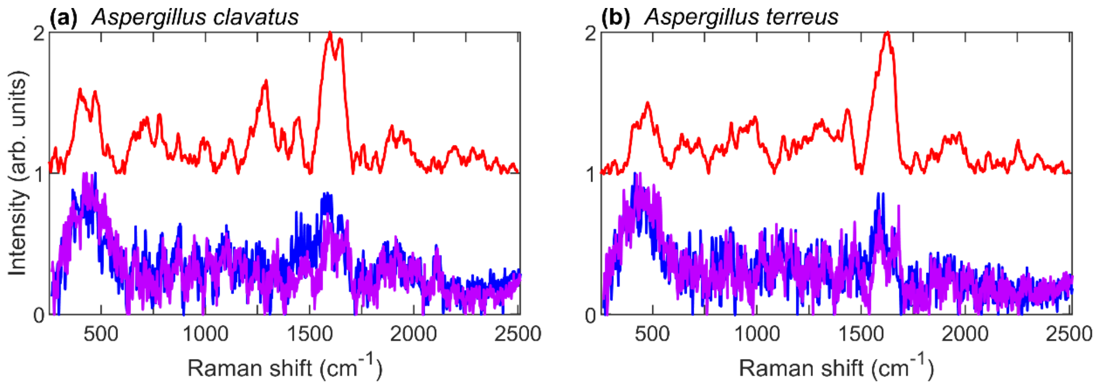

Figure S2-1. Single spore Raman spectra. The blue and purple curves correspond to Asymmetric Least-Squares (AsLS) background-subtracted raw spectra excited by two slightly different frequencies, while the red curves correspond to the reconstructed, pure Raman spectra obtained through the SERDS protocol outlined in the Supplementary Information Appendix S1.

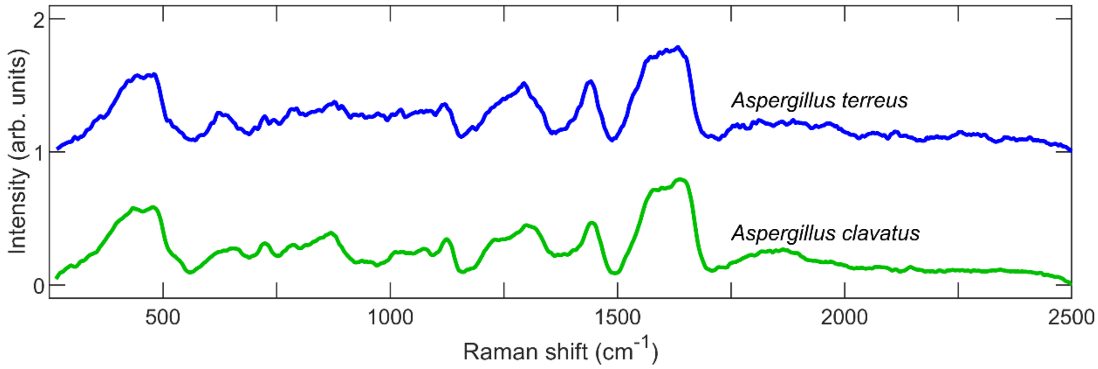

Figure S2-2. Pure Raman spectra retrieved from the SERDS protocol outlined in the Supplementary Information Appendix S1. Each spectrum is the average of the SERDS spectra retrieved from $n = 50$ and $n = 10$ individual spores of *A. clavatus* and *A. terreus*, respectively.
